# Study of trijet production in proton-proton collisions at different energies

**DOI:** 10.1038/s41598-025-11867-1

**Published:** 2025-07-22

**Authors:** M. A. Mahmoud, M. Gamal, S. El-Sharkawy, N. N. Abd Allah, K. Mohamed

**Affiliations:** 1 Physics Department, Faculty of Science, Fayoum University, El-Fayoum, 63514 Egypt; 2 Center for High Energy Physics (CHEP-FU), Fayoum University, El-Fayoum, 63514 Egypt; 3 Physics Department, Faculty of Science, Sohag University, Sohâg, 82524 Egypt

**Keywords:** pp, QCD, Event generators, Trijet, Forward dijet, Central jet, PYTHIA8, HERWIG++, SHERPA, Particle physics, Experimental particle physics

## Abstract

The 3-jet events produced from proton-proton collisions at center of mass energies $\sqrt{s}=$13, 13.6, 14, 20, and 27 TeV were considered. These energies match those of the Large Hadron Collider, both present and maybe future energies, we require one jet to exist in the central pseudorapidity region $(|\eta | < 2.0)$ and two jets in a forward (same hemisphere) region $(|\eta | >2.0)$. We compare the predictions of PYTHIA, HERWIG++, and SHERPA, among other Monte Carlo Event Generators. We examine the distributions of the jets’ transverse momentum, jet multiplicity, azimuthal angle, and pseudorapidity. We also examine the distribution of azimuthal angle differences between the center jet and the forward dijet system. We compared PYTHIA8 predictions with CMS data at $\sqrt{s} = 7$TeV for dijet azimuthal angle differences ($\Delta \phi$). The cross section rises with $\Delta \phi$ and peaks near $\pi$, reflecting momentum conservation in hard scattering. The relevant Rivet analysis code was used to obtain the results. Three event generators are examined in the study, and the results show both similarities and variations in their predictions. All three generators agree at low jet multiplicities. There are differences at greater multiplicities when the collision energies rise: HERWIG’s predictions rise while PYTHIA8’s fall in comparison to SHERPA. Other investigations show similar patterns, with generators agreeing in some regions (like central $\eta$ distributions) but diverging in others (like high/low $\eta$ or high $\Delta \phi _{dijet}$). These differences are likely due to the underlying theoretical models used by each generator. The results obtained emphasize the significance of generator-specific tuning for precision QCD research and new physics investigations at existing and future colliders.

## Introduction

Research on particle collisions has significantly advanced our understanding of the strong interaction in quantum chromodynamics (QCD) and enriched our knowledge of the parton distribution functions (PDFs) of the proton^1,2^. Particle physics’ standard model (SM) includes a number of well-documented shortcomings^3^. The hierarchy problem, dark matter, the nonzero neutrino masses, the matter-antimatter asymmetry of the universe, and the proliferating quarks and leptons are on this list^4^. A large program of high-energy collider searches for physics outside the Standard Model has been sparked by these shortcomings. Large cross section creation of new particles related to quarks and/or gluons makes them particularly appealing for searches at hadron colliders such as the CERN LHC^5^. Numerous searches at previous and contemporary colliders have been conducted for novel particles decaying to two hadronic jets (dijets) ^6–16^.

High-energy particle interactions are characterized by collimated streams of hadrons, Jets in quantum chromodynamics (QCD), the theory of strong interactions, can be understood as products of parton fragmentation produced within a scattering process. Two fundamental phases are observable in collisions with high energy particles. Partons with high transverse momentum $(p_T)$ in the perturbative phase are produced at a scale *Q* by a hard scattering process. In QCD, a perturbative expansion describes this phase. These partons form quark-antiquark pairs emit more gluons as they go into the second (non-perturbative) phase. The interaction between the underlying event and the hadronization process results in the non-perturbative jet growth^17^. Despite these consequences, highly collimated particle sprays (jets) exist in the final state and are collectively called hadron jets. Hadronization and the underlying event both have distinct effects that change significantly with the jet radius parameter and become more noticeable at low $p_T$. Phenomenological models that are set to the data are used to account for them^18–20^.

High-energy particle collisions create jets, or collimated sprays of hadrons, in large quantities. Jets continue to be important objects of study even at the high energy obtained at the Large Hadron Collider (LHC). Furthermore, the fact that they exist is evidence of violent processes occurring at energies far greater than that of the colliding particles—between tens of GeV and half of their total original energy. Jets may be used for investigating Quantum Chromodynamics and the Standard Model (SM) in general as they are really QCD objects. Jets are employed in the study of the flavor sector of QCD and other aspects of the strong interaction, including measurements of the strong coupling^21–23^. Jets are thought to be useful instruments for investigating the properties of strong interactions and proton quantity. Thus, one of the best means of studying perturbative quantum chromodynamics (QCD) are the jet production processes at the Large Hadron Collider (LHC).

Jets are also created from hadronic decays of heavy particles, such the top quark decaying into three jets or the Higgs boson or vector bosons, which decays into two jets each. This is due to the possibility that jets originate from sources other than short-range quark-gluon interactions. In instances where a monojet recoils against the absent energy, even the dark matter and other “dimensions” are searched for^24^.

According to the study^25^, the trijet production process at the LHC is quite interesting because it can test parton shower characteristics and provide insights about the dynamics of the transverse momentum dependence of proton components. It also allows one to study the properties of ITMD factorization and, last but not least, it shows the real emission contribution to dijet formation at NLO^26^. The study events with two forward jets, and also on a third jet produced in the central region of rapidity, allowing for inclusive radiation in the remaining areas of the detectors.

In our work, we present predictions for a new investigation of the forward dijet and one center jet using several Monte Carlo event generators. The paper is structured as follows: In the following section, we will briefly outline the Monte Carlo event generator used in our calculations. In section “﻿Results and discussion”, we outline kinematic cuts and discuss their results. Section “Conclusions” concludes with a summary. We now study the behavior of these generators at greater energy, $\sqrt{s} = 7$ TeV, $\sqrt{s} = 13$ TeV, $\sqrt{s} = 13.6$ TeV, $\sqrt{s} = 14$ TeV $\sqrt{s} = 20$ TeV and $\sqrt{s} = 27$ TeV, where future runs of the LHC might be operated.

## Event selection

The study’s data was collected using the event generators PYTHIA8, Herwig7, and Sherpa. To allow direct comparisons of simulation outcomes, the same procedures and criteria for event generators were employed.

Using the anti-$k_{T}$^27^ approach on the distance parameter $R = 0.4$, particles are clustered into jets having $p_T > 20$ GeV and pseudorapidity $|\eta | < 4.7$ in all samples. Furthermore, we need one jet in the center pseudorapidity region $(|\eta _{c}| < 2.0)$ and two jets in forward region $(|\eta _{f1,f2}| > 2.0)$, both in identical pseudorapidity hemisphere $(\eta _{f1}. \eta _{f2} > 0)$. Lastly, the leading jet needs $p_T > 35$ GeV. These predictions were obtained by using $2 \rightarrow 2$ hard scattering processes complemented with parton showers to account for the third jet.

Some experimental data from CMS for dijet azimuthal angle differences ($\Delta \phi$)^28^ were compared the PYTHIA8 and HERWIG at energy 7 TeV with cuts $80<p_{T}^\textrm{leading}<110\,\hbox {GeV}$, $110<p_{T}^\textrm{leading}<140\,\hbox {GeV}$, and $|\eta | < 1.1$.

We use the NNPDF2.3 NLO parton distribution functions^29^ via LHAPDF6. Using appropriate Rivet^30^ analysis code for this study, the produced events from Monte Carlo Events Generators (MCEGs) were processed to provide results.

## Monte Carlo generator theory

Using Monte Carlo (MC) event generator is a method to simulate the kinds of events that should be produced at the LHC in proton-proton collisions. They also provide helpful guidance for selecting or developing existing experiments as well as building new ones^31,32^. One may simulate proton-proton collision events and the particles generated in such collisions using several types of Monte Carlo (MC) event generators. Due of their prevalence, PYTHIA8, HERWIG7, and SHERPA will be the main focus in this study. These generators are employed in many different experimental tasks, including fiducial cross section extrapolation to the full phase space, detector acceptances and selection efficiency evaluation, and calibration of object reconstruction algorithms.

Additionally, during the past 10 years, Monte Carlo (MC) event generators have become more and more popular as a technique for accurate predictions of scattering cross section, event topology, and differential distribution. Now, because of the ongoing introduction of higher-order perturbative corrections, they give state-of-the-art theoretical calculations that allow accurate analyses and data interpretation, mainly in QCD but also in QED and the electroweak sector. They may be used to accurately model Standard model production processes and define every New Physics signal due to their high degree of automation. A crucial component of collider-based particle physics, from real Standard Model observations to searches for unknown things are Monte-Carlo (MC) event generators^33^.

### PYTHIA8

A famous scientific code library for modeling events involving a high energy collisions of particles where quantum chromodynamics (QCD) governs the strong nuclear force is PYTHIA 8.3^34^. The Lund string hadronization model^35^ provides his basis. Several libraries, including a hard process, models for multiple parton interactions (MPIs), initial-state radiation (ISR), final-state radiation (FSR), and particle decays, are included in this tool to help in generating of events in a high energy collisions^36^. It is also used to define physics by comparing it with existing data, and to predict new physics. PYTHIA’s code was first written in FORTRAN, however it has now been updated to C. These days, PYTHIA simulates dark matter annihilation into standard model particles, gamma-hadron interactions, hadron-hadron collisions, and lepton-lepton collisions^37^. Large Hadron Collider (LHC) experimental partnerships now comprise the biggest user group, although the software is also utilized for several other phenomenological or experimental research in particle, nuclear, and astrophysics.

#### The Lund String model

In the model, the probability represent a string breaking into a state that has n hadrons. Specifically, the probability $d\mathcal {P}_{n}$. of getting a state including n hadrons with (simplistically, equivalent) mass m and energy-momentum vectors $p_{1}.... p_{n}$. is provided by^35^

1 $$\begin{aligned} d\mathcal {P}_{n}\propto \Biggl \{\prod _{i=1}^{n} [Nd^{2}p_{i}\delta (p^{2}_{i}-m^{2})]\delta ^{(2)}(\sum p_{i}-P_{tot})\Biggl \}exp(-bA) \end{aligned}$$

The expression in curly parenthesis is called a phase space factor, where *N*, the dimensionless constant, determines the weighting of states with different meson counts. The term *bA* in the exponent represents the imaginary part of the massless string’s action, which leads to the string’s decay and finite lifetime. Before the string splits apart, *A* measures the space-time area of the string, and *b* is a constant. Traditionally, the square of the string tension $\kappa$ is used to scale the area *A*:

2 $$\begin{aligned} A \equiv \mathcal {A}\kappa ^{2} \end{aligned}$$

where the area in space and time is denoted by $\mathcal {A}$. The result in Eq. (1) can be obtained by a Monte Carlo simulation by generating the mesons iteratively, starting from one of the string ends, and using a proportion z of the remaining energy for each meson. The probability distribution or splitting function determines the proper z-value for each step^38^:

3 $$\begin{aligned} f(z) = N \frac{(1 - z)^{a}}{z}e^{(-bm^{2}/z)} \end{aligned}$$

Here, the normalization requirement $\int f(z) dz= 1$ links the constant *a* to *N* and *b*. The pairs’ production points will be situated in space-time around a hyperbola, and their usual proper times will be established by^38^:

4 $$\begin{aligned} \langle \tau \rangle =\frac{1+a}{b\kappa ^{2}} \end{aligned}$$

Additionally, this timescale and particle multiplicity are related by^38^:

5 $$\begin{aligned} \frac{dN}{dy}\sim \sqrt{\langle \tau \rangle }\frac{\kappa }{m}=\sqrt{\frac{1+a}{bm^{2}}} \end{aligned}$$

### HERWIG7

Various high-energy collisions (lepton-lepton, hadron-hadron) may be generated using the flexible Monte Carlo event generator Herwig7^39^. It provides significant scattering processes for in-depth investigation. Both initial- and final-state QCD radiation (including color coherence effects) is accurately simulated by the parton-shower process, with a focus on modeling radiation from heavy particles. An eikonal model simulates the activity that occurs in addition to the main interaction, or the underlying event. In order to describe hadron production from partons in the parton shower, Herwig7 applies the cluster hadronization model. Hadron decays are modeled whenever possible using matrix elements, such as spin correlations and off-shell effects.

#### Cluster Hadronization model

Herwig uses the cluster model^40^ to simulate hadronization. It is based on the angular-ordered parton shower’s color preconfinement property^41^. This model’s characteristics include being local in parton color and independent of the collision’s center of mass energy and hard process^40,42^. At the end of the parton shower, the remaining gluons are first split into quark-antiquark pairs nonperturbatively in the cluster hadronization model. Following an isotropic decay of the gluon into any of the quark flavors that are available, the event is limited to color-connected (di)quarks and anti-(di)quarks. The color singlets generated by these color-connected parton pairs cluster together, with the momentum given by the sum of the momenta of all the partons. The cluster model is predicated on the idea that the clusters may be thought of as very excited hadron resonances that decay into the hadrons that were seen based on phase space. Thus, these heavy clusters broke into lighter clusters before they decay. When a cluster splits into two clusters due to a mass, M, then

6 $$\begin{aligned} M^{Cl_{pow}} \ge {Cl_{max}}^{Cl_{pow}} + (m_1 + m_2)^{Cl_{pow}} \end{aligned}$$

where the masses of the cluster’s a component partons are denoted by $m_{1,2}$, and the model’s parameters are $Cl_{max}$ and $Cl_{pow}.$

The decay of the cluster into a pair of hadrons is the last step in the cluster hadronization model. For a given cluster $(q, \bar{q})$, a quark-antiquark or diquark-antidiquark pair $(q_{1}, \bar{q}_{2})$ is obtained from the vacuum, and a pair of hadrons with flavours $(q_{1}, \bar{q})$ and $(q, \bar{q}_{2})$ are produced. Based on the available phase space, spin, and hadrons’ flavor, the applicable hadrons are determined from all potential hadrons with the appropriate flavor. While the fundamental approach used by all cluster models is the same, there are some fundamental variances. Herwig7 is used to implement the approaches of Ref^43^, the original approaches are in Ref.^40,44,45^, and a new variation that addresses the issue of the low rate of baryon production is described in Ref.^43^. Thus, the weight for the production of the hadrons $a(q_{1}, \bar{q})$ and $b(q, \bar{q}_{2})$ is given, just like in^39^.

7 $$\begin{aligned} W(a_{(q_{1}, \bar{q})}, b_{(q, \bar{q}_{2})}|q_{1}, \bar{q}_{2}) = P_{q}w_{a}w_{b}s_{a}s_{b}p^{*}_{a,b} \end{aligned}$$

where $p^{*}_{a,b}$ is the momentum of the hadrons in the rest frame of the decaying cluster, $w_{a,b}$ are the weights for the production of individual hadrons, and $s_{a,b}$ are the suppression factors for the hadrons, which allow the production rates of individual meson multiplets, singlet, and decuplet baryons to be adjusted. where $P_q$ is the production weight of the provided pair of quarks and antiquarks or diquarks and antidiquarks.

### SHERPA

A multipurpose event generator framework for high-energy particle collisions is SHERPA^33^. It contains all the implementations needed for hadron-hadron, lepton-hadron, and lepton-lepton scattering events at colliders, in a factorized and probabilistic approach. The latter involves parameters to consider into account, the physics model, and the initial beam configuration.

The two integrated tree-level matrix element generators in SHERPA are COMIX^46^ and AMEGIC^47^. They have usage in the decay of heavy resonances like W, Z, or Higgs bosons or top quarks, as well as in the modeling of parton-level processes both within and beyond of the Standard Model. Both involve algorithms for removing infrared divergences in calculations at next-to-leading order (NLO) in QCD and the electroweak theory, as well as automated techniques for effective phase-space integration. SHERPA assesses virtual corrections at NLO accuracy by utilizing interfaces to specialized one-loop providers like BLACKHAT, OPENLOOPS, and RECOLA.

#### Cluster fragmentation in AHADIC

The AHADIC module in SHERPA implements the cluster concept^33^. At the conclusion of the perturbative phase, non-perturbative gluon decays give rise to the formation of quark/anti-quark. We should note that the word “quark” is used here to refer to both quarks and diquarks. The phase space that is open to them, which is determined by their constituent masses, and additional suppression weights that are distinct to each flavor influence their choice^33^.

The splitting kinematics are in a dipole frame, where the spectator object provides the required recoil and the gluons stay massless^33^.

AHADIC forms color-neutral clusters by combining color-connected quarks and anti-quarks after the gluon decays. Depending on their mass, these clusters decay into hadrons or into other clusters. For all types of decay, flavor pairings need to be recreated with the same suppression weights as for gluon splittings. One can determine whether a cluster is too light and will decay into two hadrons by comparing the cluster mass $M_c$ with a critical mass $M_{crit}$ that is determined by combining the masses of the lightest and heaviest hadron pairs, $M_{-}$ and $M_{+}$, that might emerge in the cluster decay^33^.

8 $$\begin{aligned} M_{crit} = M_{-} (1 - \kappa ) + M_{+} \kappa \end{aligned}$$

with the off-set parameter which is $\kappa$. If the quark cluster $q_1\bar{q}_{2}$ is less than $M_{c}$, it will decay into two hadrons. Each decay channel $C\rightarrow h_1h_2$ has relative probabilities that are determined by^33^:

9 $$\begin{aligned}&\mathcal {P}(C\rightarrow h_1h_2) \nonumber \\&\quad =\mathcal {P}_{q}|\psi _{1}(q_{1}\bar{q})|^{2}|\psi _{2}(q\bar{q}_{2})|^{2}\mathcal {P}_{multi}\sqrt{(M_{c}^{2}-m_{1}^{2}-m_{2}^{2})^{2}-4m_{1}^{2}m_{2}^{2}}8\pi M_{c}^{2}\Biggl (\frac{(m_{1}+m_{2})^{2}}{M_{c}^{2}}\Biggl )^{\chi } \end{aligned}$$

## Results and discussion

**Fig. 1:**
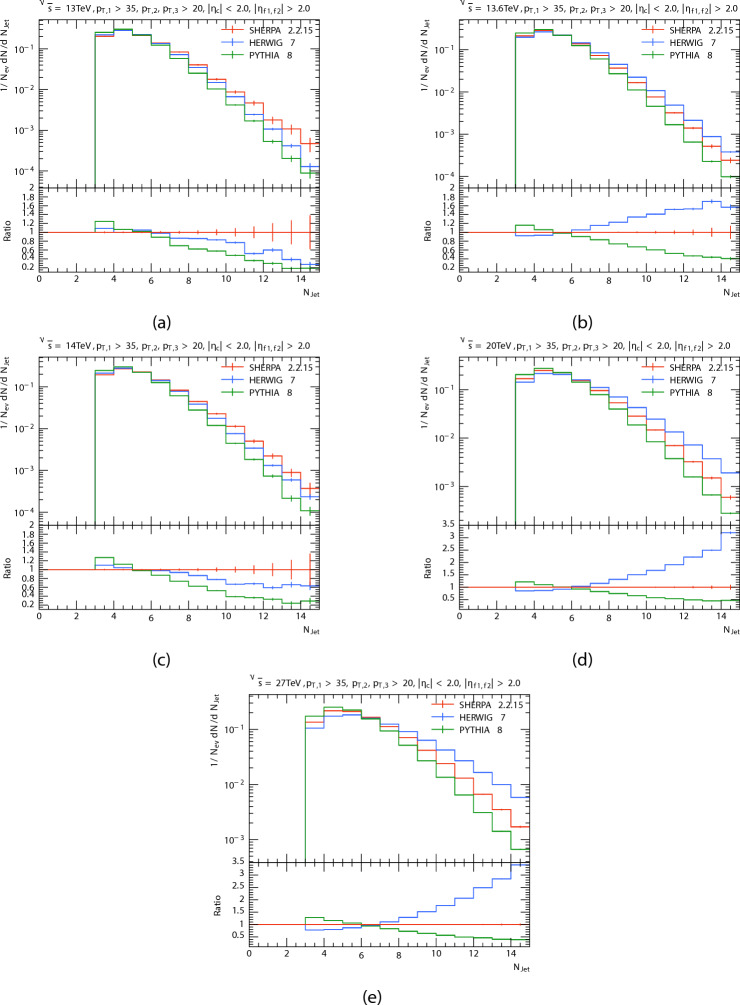
(**a**–**e**) Are shown jet multiplicity distributions at 13, 13.6, 14, 20 and 27 TeV respectively, after the procedure, compared PYTHIA ,HERWIG with SHERPA predictions.

The multiplicity of three jets, two in a forward region $(|\eta _{f1,f2}| > 2.0)$, and one in the central region $(|\eta _{c}| < 2.0)$ in P-P collisions at energies 13 TeV, 13.6 TeV, 14 TeV, 20 TeV, and 27 TeV is shown at Fig. 1. The ratio of PYTHIA and HERWIG predictions to SHERPA predictions is displayed in the bottom panel. And the Table 1shows summary of average jet multiplicity $\langle N_{\text {jets}} \rangle$, standard deviation, and maximum multiplicity for each generator at various center-of-mass energies. For a minimum of three jets, the figures indicate that the width of the distributions rises with increasing C.M. energy, and for higher energies, bigger values of $N_{jet}$ are obtained as expected. The expected number of events drops with rising $N_{jet}$ for $N_{jet}>6$. PYTHIA produces a little greater relative number of large density events for all energies than HERWIG and SHERPA do for low multiplicity$(N_{jet}<5)$. It seems that for the $N_{jet}$ distribution, predictions of PYTHIA8, HERWIG, and SHERPA are matching at low multipicity but, at high multipicity the PYTHIA8 decreased in relation to the SHERPA , and the opposite happened for HERWIG for all energies, But when the energy increases, the increase in the predictions of the HERWIG increases more than that of the PYTHIA8 and SHERPA. We can see that, when the center-of-mass energies increased the difference between the simulated data increased at high multipicity. This discrepancy is probably caused by variations in the hadronization processes used in each generator and parton shower models.

**Table 1:** Summary of average jet multiplicity $\langle N_{\text {jets}} \rangle$, standard deviation, and maximum multiplicity for each generator at various center-of-mass energies.

$\sqrt{s}$ (TeV)	Generator	$\langle N_{\text {jets}} \rangle$	$\sigma _{N_{\text {jets}}}$	$N_{\text {jets,max}}$
13.0	PYTHIA8	5.081	1.451	4.50898
13.0	HERWIG	5.275	1.564	4.50355
13.0	SHERPA	5.403	1.673	4.50355
13.6	PYTHIA8	5.116	1.471	4.50898
13.6	HERWIG	5.488	1.714	4.52081
13.6	SHERPA	5.323	1.601	4.52081
14.0	PYTHIA8	5.131	1.48	4.50641
14.0	HERWIG	5.355	1.619	4.50641
14.0	SHERPA	5.496	1.714	4.51726
20.0	PYTHIA8	5.394	1.639	4.52081
20.0	HERWIG	6.064	2.058	4.50898
20.0	SHERPA	5.69	1.812	4.50898
27.0	PYTHIA8	5.65	1.791	4.50197
27.0	HERWIG	6.67	2.378	5.50197
27.0	SHERPA	6.069	2.025	4.50197

**Table 2:** Mean transverse momentum $\langle p_T \rangle$ of leading, subleading, and third jets at different energies for each generator. Uncertainties represent statistical errors.

$\sqrt{s}$ (TeV)	Jet rank	PYTHIA8 (GeV)	HERWIG (GeV)	SHERPA (GeV)
13.0	Leading	54.18 ± 0.5804	51.81 ± 0.4611	56.98 ± 2.409
13.0	Sublead.	37.67 ± 0.4009	36.48 ± 0.3391	40.82 ± 1.787
13.0	Third	27.662 ± 0.1422	27.41 ± 0.1252	26.81 ± 0.4364
13.6	Leading	54.17 ± 0.1818	52.11 ± 0.1592	57.16 ± 0.6295
13.6	Sublead.	37.63 ± 0.1254	36.61 ± 0.1154	41.08 ± 0.4625
13.6	Third	27.63 ± 0.04452	27.4 ± 0.04217	26.93 ± 0.1157
14.0	Leading	54.19 ± 0.5697	51.78 ± 0.4525	56.9 ± 1.918
14.0	Sublead.	37.64 ± 0.3934	36.5 ± 0.332	40.98 ± 1.41
14.0	Third	27.62 ± 0.1394	27.43 ± 0.1231	26.9 ± 0.357
20.0	Leading	54.4 ± 0.1675	51.88 ± 0.1399	57.21 ± 0.717
20.0	Sublead.	37.9 ± 0.115	36.6 ± 0.1017	41.3 ± 0.525
20.0	Third	27.83 ± 0.0416	27.44 ± 0.03762	27.06 ± 0.1346
27.0	Leading	54.5 ± 0.1577	51.51 ± 0.1237	57.26 ± 0.5181
27.0	Sublead.	38.07 ± 0.1081	36.45 ± 0.09042	41.43 ± 0.3775
27.0	Third	27.97 ± 0.03956	27.44 ± 0.03366	27.19 ± 0.09813

**Fig. 2:**
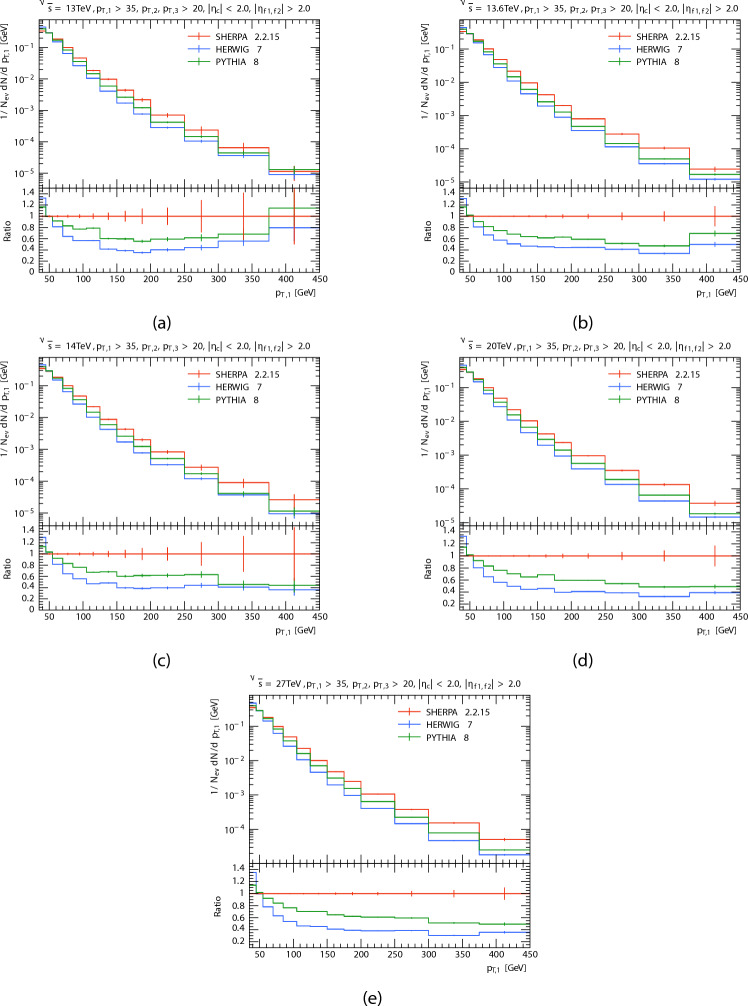
Leading jets’ transverse momentum $p_T$ distributions. At energies of 13, 13.6, 14, 20, and 27 TeV.

**Fig. 3:**
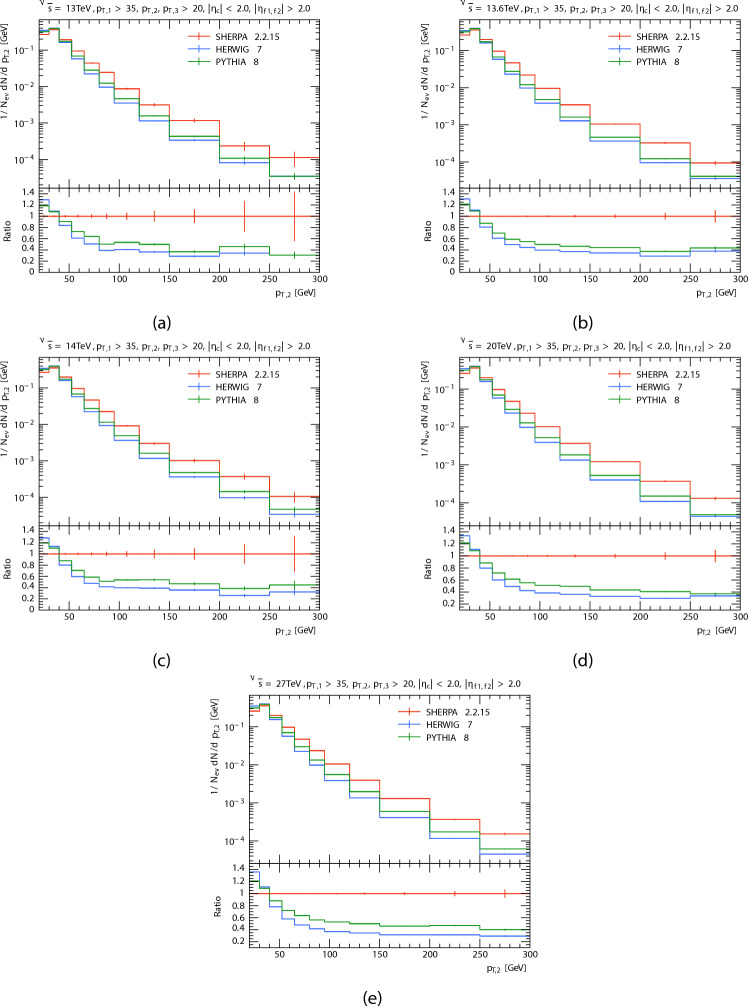
Subleading jets’ transverse momentum $p_T$ distributions. At energies of 13, 13.6, 14, 20, and 27 TeV.

**Fig. 4:**
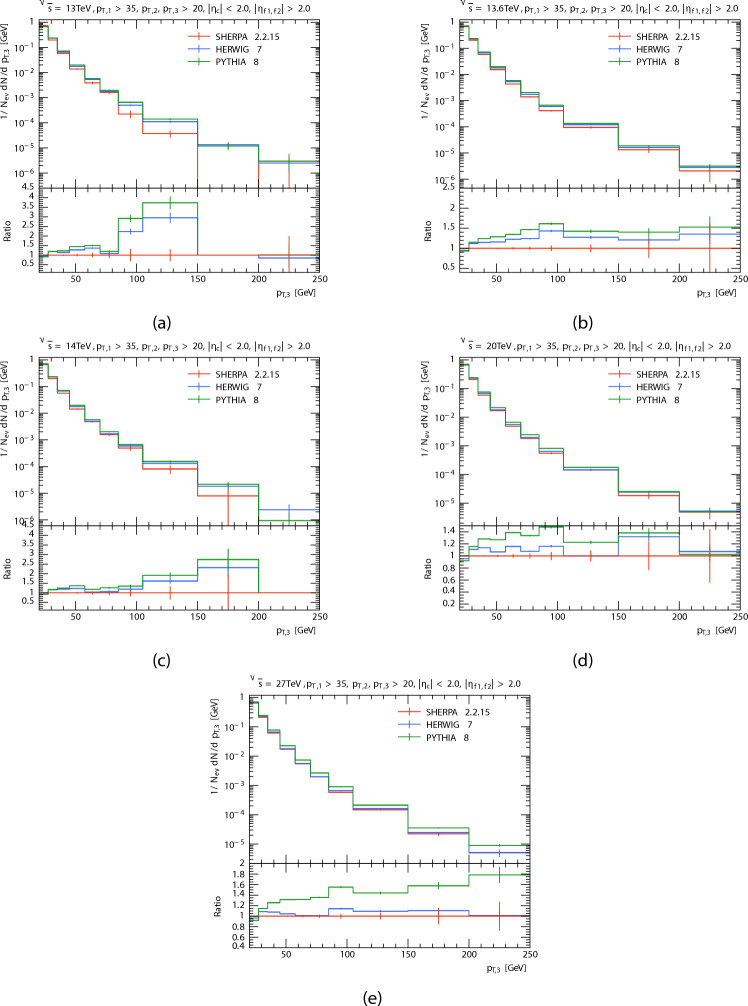
Third jets’ transverse momentum $p_T$ distributions. At energies of 13, 13.6, 14, 20, and 27 TeV.

The plots in Figs. 2, 3 and 4 show the obtained $p_T$ spectra distributions for the three leading jets, in P-P collision at $\sqrt{s}=13\,\hbox {TeV}$, 13.6 TeV, 14 TeV, 20 TeV, and 27 TeV from PYTHIA8, HERWIG, and SHERPA and their corresponding ratio. And the Table 2 shows the mean transverse momentum $\langle p_T \rangle$ of leading, subleading, and third jets at different energies for each generator. It is clear that as momentum rises the anticipated number of events decreases, this because there are phase limitations in space and decreasing probability of finding high momentum partons within protons. For leading and subleading jets. It seems to both generators (PYTHIA8 and HERWIG) predict a higher than the SHERPA predicted at low $p_T$. At high $p_T$, PYTHIA’s prediction is higher than both other event generators, and as energy increases, SHERPA’s prediction increases and the predictions of both generators are less than the predictions of SHERPA. It is seen that both generators predict a similar yield. With the the expected number of events of PYTHIA8 slightly higher than HERWIG. The opposite occurs in the case of the third jet, PYTHIA and HERWIG predictions are somewhat similar and higher than SHERPA’s prediction. At high $p_T$, all event generator predictions are similar at energy 13 TeV. It is seen that a similar yield for the the expected number of events of SHERPA with HERWIG when the center-of-mass energies increased. This occurred as a result of data from SHERPA showing distinct behavior at high $p_T$. Disparities in the underlying event modeling and matrix element-parton shower merging techniques may be the cause of this behavior.

**Fig. 5:**
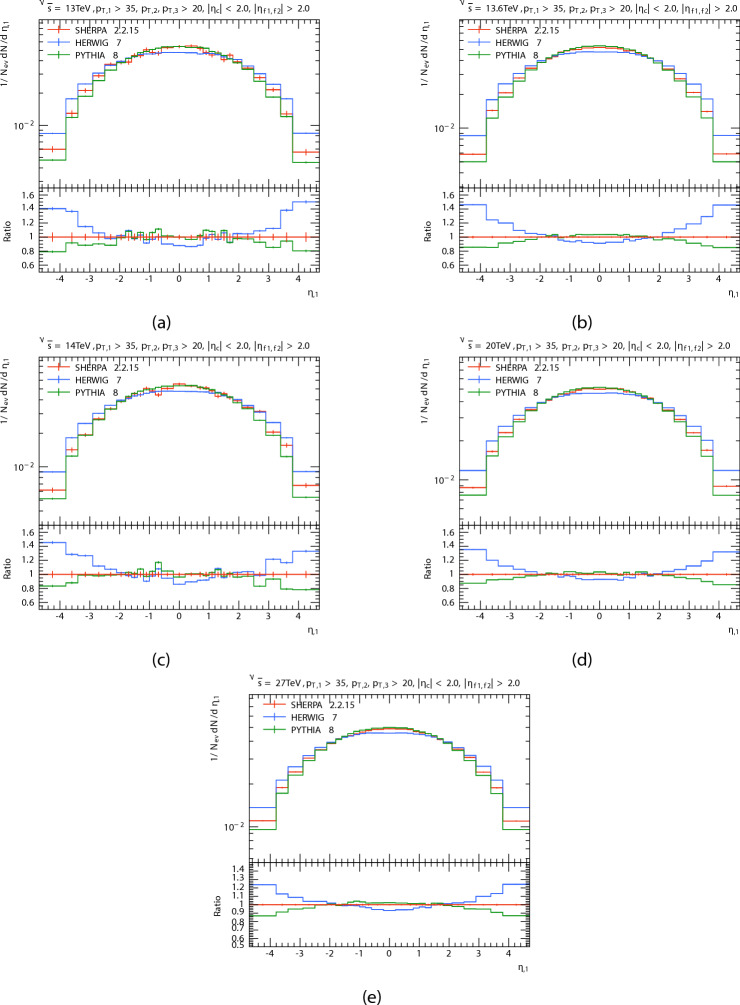
Leading jets’ pseudorapidity $\eta$ distributions. At energies of 13, 13.6, 14, 20, and 27 TeV.

**Fig. 6:**
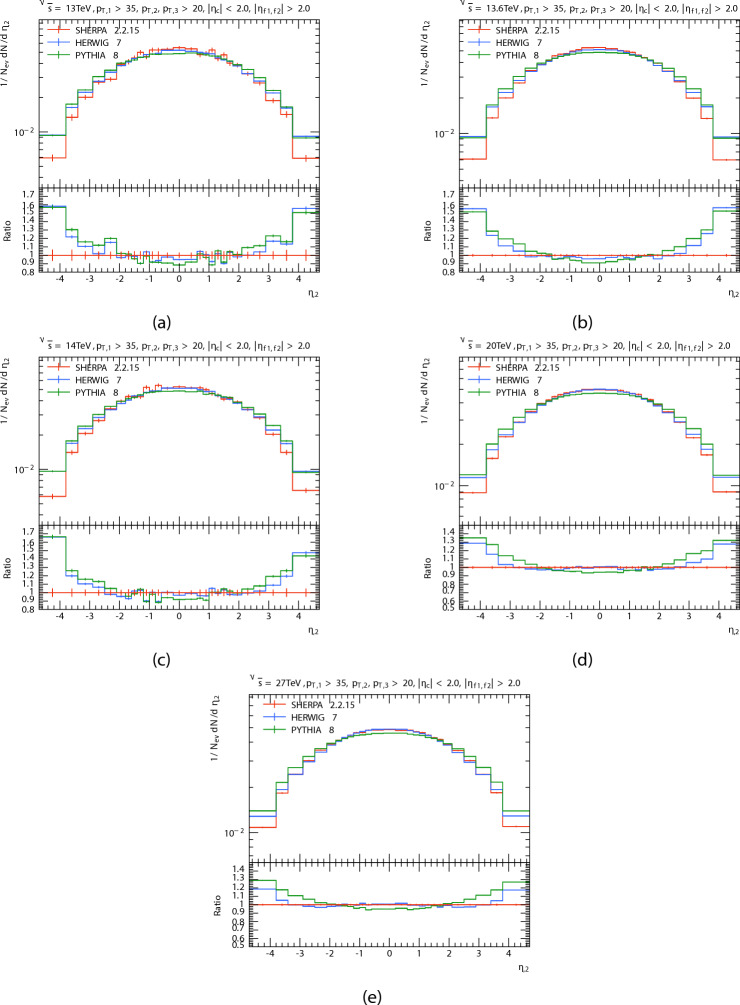
Subleading jets’ pseudorapidity $\eta$ distributions. At energies of 13, 13.6, 14, 20, and 27 TeV.

**Fig. 7:**
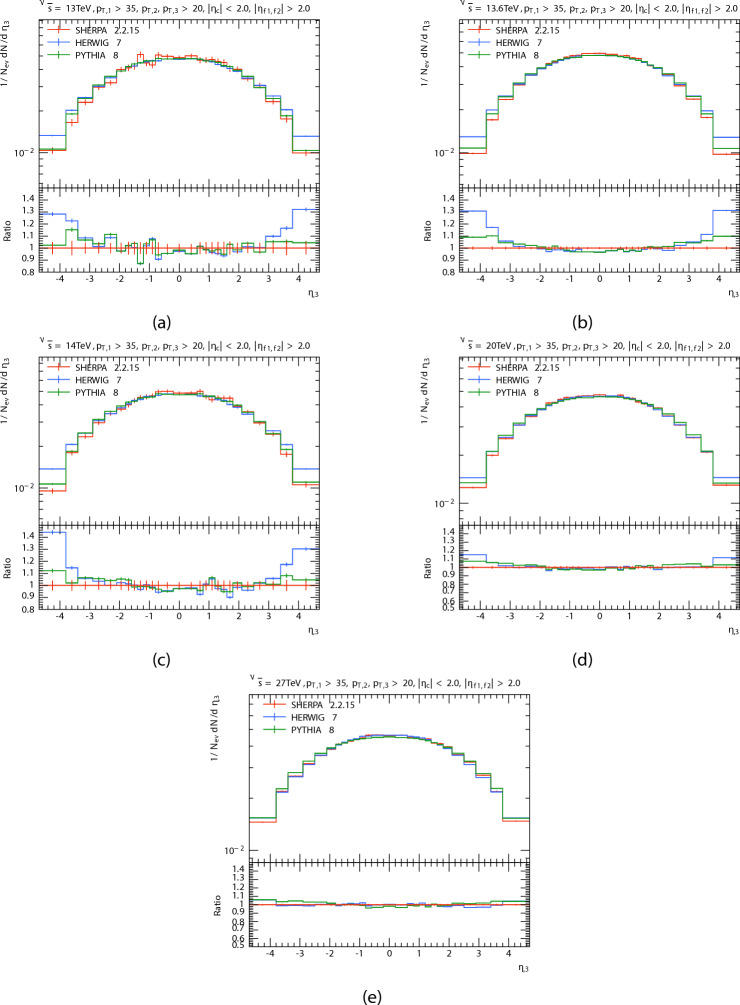
Third jets’ pseudorapidity $\eta$ distributions. At energies of 13, 13.6, 14, 20, and 27 TeV.

The pseudorapidity distribution $\eta$ of the three leading jets are presented in Figs. 5, 6 and 7 in P-P collision at $\sqrt{s}=$13 TeV, 13.6 TeV, 20 TeV, 14 TeV and 27 TeV from PYTHIA8, HERWIG, and SHERPA and their corresponding ratio. And the Table 3 shows the pseudorapidity $\langle \eta \rangle$ of leading, subleading, and third jets at different energies for each generator.

The pseudorapidity distribution in the central region $(- 2.0< \eta < 2.0)$ can be seen in this figure as quasi-flat form that is consistent with theory, confirming the generators’ dependability in this kinematic range. For theoretical estimation, the presence of plateaus in the rapidity or pseudorapidity distribution is important^48^. It is also observed that the multiplicity of the jet is greater at the central regions than at the forward regions. This is because the central regions have more efficient energy deposition and higher density parton interactions. It is also observed that the multiplicity of the jet is greater at the central regions than at the forward regions. This is because the central regions have more efficient energy deposition and higher density parton interactions. It seems that for the $\eta _{1}$ distribution, predictions of PYTHIA8, HERWIG, and SHERPA are in a good agreement at central region with a slight decrease in the predictions of HERWIG in the leading jet compared to the predictions of the event generators and in PYTHIA in the subleading jet. but, at the PYTHIA8 decreased in relation to the SHERPA at high and low $\eta _{1}$, and the opposite happened for HERWIG for all energies. when the energy increases,the predictions of the HERWIG are close to the predictions of the SHERPA, and the pseudorapidity distribution $\eta _{2}$ of subleading jet. It seems to both generators (PYTHIA8 and HERWIG) predict a higher than the SHERPA predicted at the forward region $\eta > 2.0$. The disparities in forward jet prediction could be due to the parton showers and their interaction with non-perturbative hadronization models.And it can be noted that all generator show good agreement at central region. We notice that the matching region increases with increasing energy. The pseudorapidity distribution $\eta _{3}$ of third jet. It seems to the matching region increases with energy greater than $\eta _{2}.$

**Table 3:** Mean pseudorapidity $\langle \eta \rangle$ of leading, subleading, and third jets at different energies for each generator. Uncertainties represent statistical errors.

$\sqrt{s}$ (TeV)	Jet Rank	PYTHIA8	HERWIG	SHERPA
13.0	Leading	0.0004383 ± 0.001189	– 0.001466 ± 0.001233	– 0.003433 ± 0.004432
13.0	Sublead.	– 0.00463 ± 0.001337	– 0.004959 ± 0.001209	– 0.0007391 ± 0.004346
13.0	Third	– 0.007741 ± 0.001379	– 0.001215 ± 0.001324	0.009214 ± 0.004788
13.6	Leading	– 0.000664 ± 0.0003742	– 0.001096 ± 0.0004147	0.0002596 ± 0.00113
13.6	Sublead.	0.0001456 ± 0.0004199	– 0.004997 ± 0.0004071	– 0.0001227 ± 0.001124
13.6	Third	– 0.001719 ± 0.0004323	– 0.002697 ± 0.0004392	– 0.000006658 ± 0.001229
14.0	Leading	– 0.006607 ± 0.001189	0.009165 ± 0.001217	0.03086 ± 0.003509
14.0	Sublead.	– 0.002896 ± 0.001321	– 0.0009181 ± 0.001196	– 0.007497 ± 0.003467
14.0	Third	– 0.001458 ± 0.001355	0.0006484 ± 0.001301	0.01836 ± 0.003786
20.0	Leading	– 0.0003033 ± 0.000357	0.0007384 ± 0.0003821	0.03086 ± 0.003509
20.0	Sublead.	0.001512 ± 0.0003966	0.0005128 ± 0.0003719	– 0.007497 ± 0.003467
20.0	Third	0.002958 ± 0.0004076	– 0.0008203 ± 0.0003978	0.01836 ± 0.003786
27.0	Leading	– 0.0002941 ± 0.0003439	0.001237 ± 0.000352	0.004695 ± 0.0009923
27.0	Sublead.	– 0.0001634 ± 0.00038	– 0.002471 ± 0.0003412	0.001714 ± 0.0009902
27.0	Third	– 0.0006238 ± 0.0003908	– 0.0007741 ± 0.0003625	0.008043 ± 0.001072

**Fig. 8:**
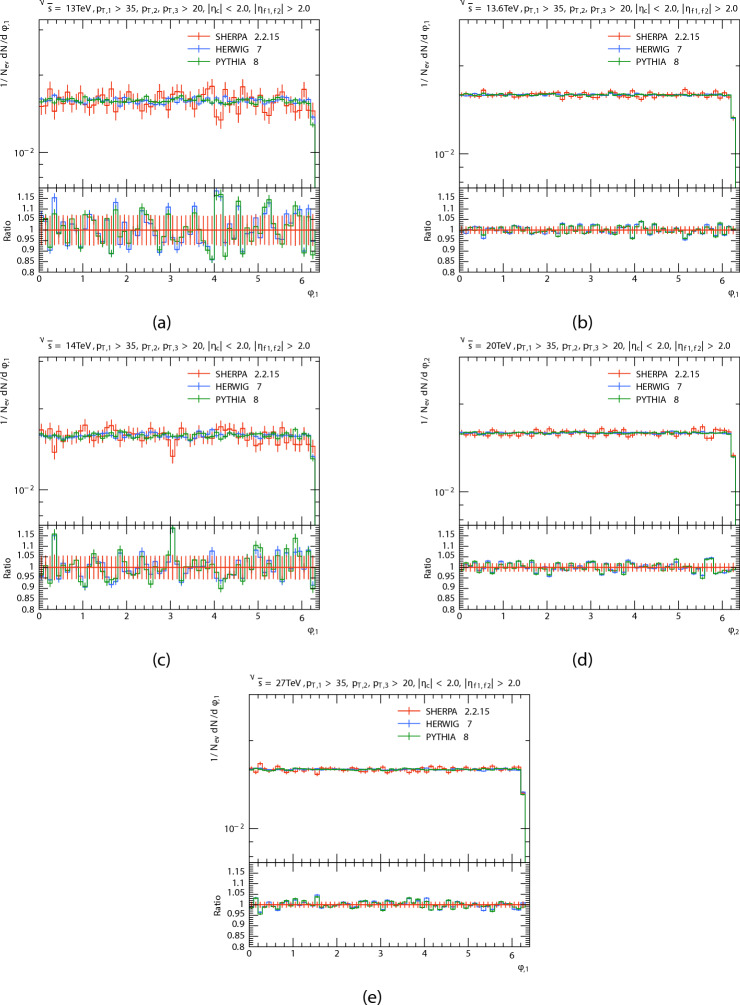
leading jets’ azimuthal angle $\phi$ distributions. At energies of 13, 13.6, 14, 20, and 27 TeV.

**Fig. 9:**
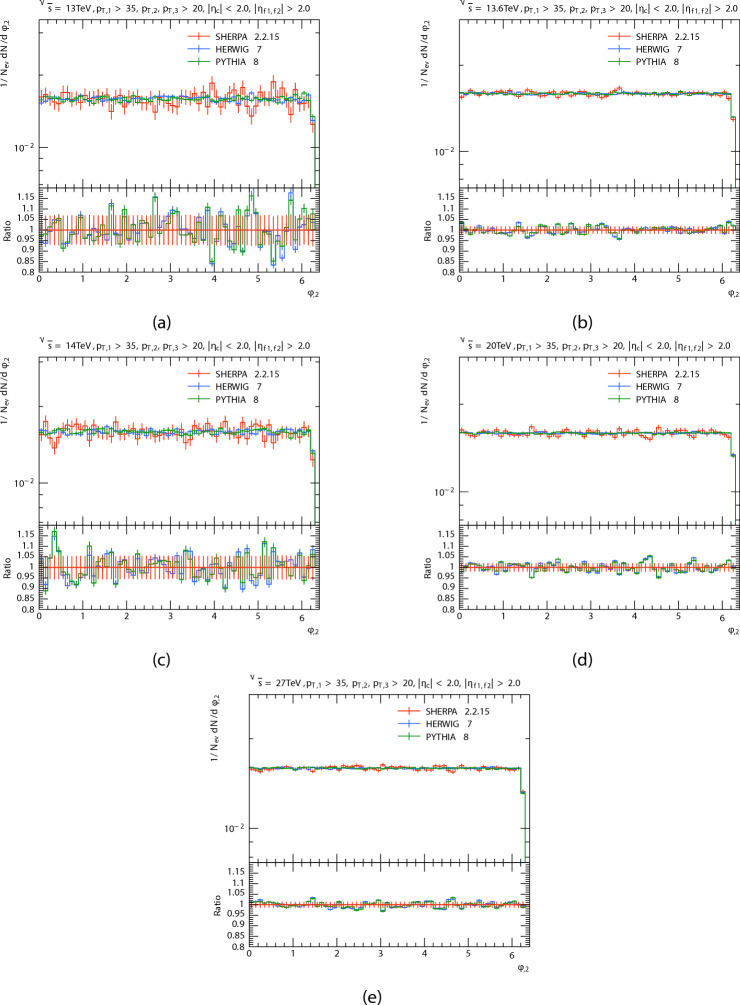
Subleading jets’ azimuthal angle $\phi$ distributions. At energies of 13, 13.6, 14, 20, and 27 TeV.

**Fig. 10:**
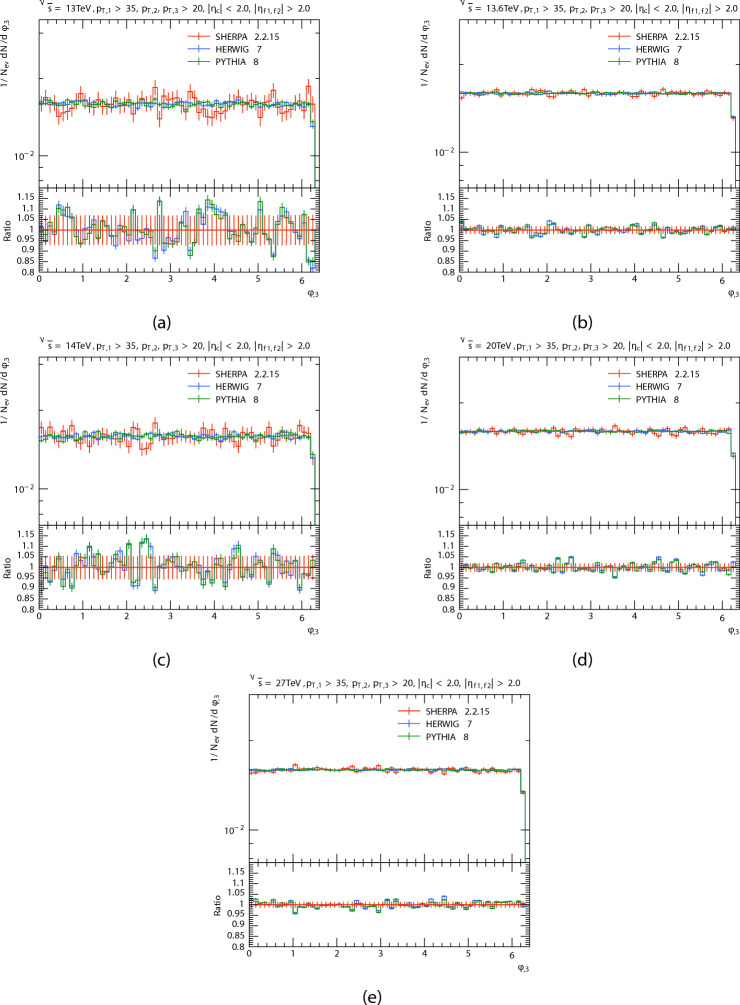
Third jets’ azimuthal angle $\phi$ distributions. At energies of 13, 13.6, 14, 20, and 27 TeV.

**Fig. 11:**
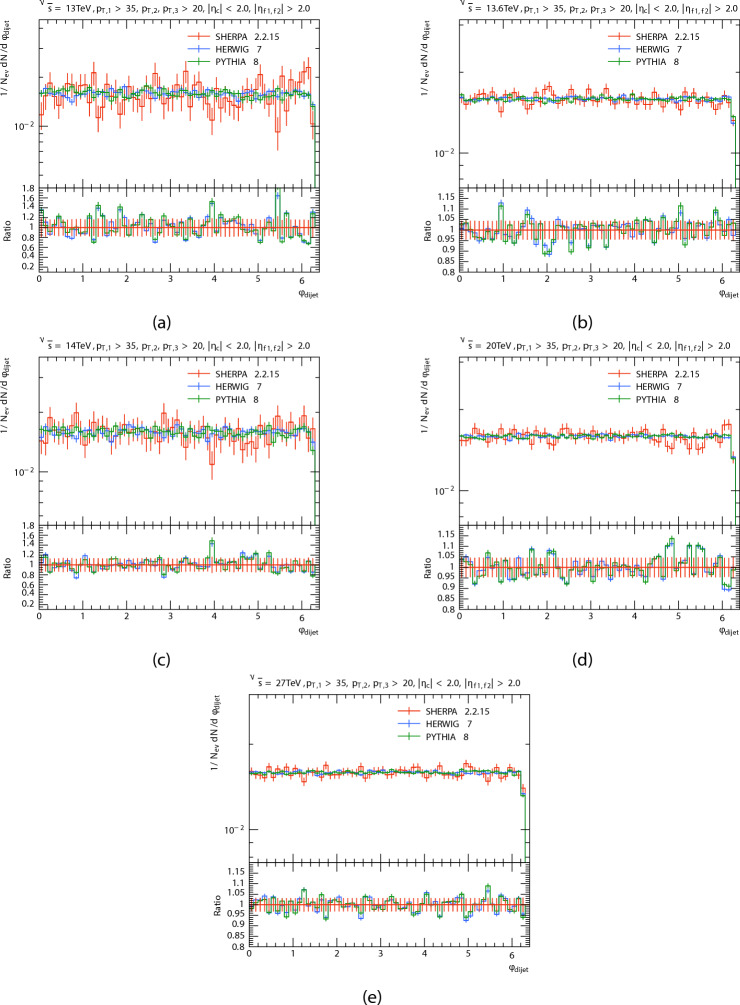
Di-jets’ azimuthal angle $\phi$ distributions. At energies of 13, 13.6, 14, 20, and 27 TeV.

The azimuthal angle distribution $\phi$ of the three leading jets and forward dijets are presented in Figs. 8, 9, 10 and 11 in proton proton collision at energis $\sqrt{s}=$13, 13.6, 14, 20, and 27 TeV from PYTHIA8, HERWIG, and SHERPA and their corresponding ratio. And the Table 4 shows the azimuthal angle $\langle \phi \rangle$ of leading, subleading, third jets, and forward dijets at different energies for each generator. The predictions have a quasi-flat distribution, there are some oscillations in the predictions that are more noticeable in SHERPA and HERWIG’s distribution throughout all regions; and there are Some agreement between PYTHIA8 and HERWIG, which can be noted, Unlike SHERPA. This oscillations, particularly in the HERWIG and SHERPA predictions, could be due to variations in how angular ordering and coherence effects are used in parton showers. As for the Dijet system, the SHERPA fluctuate is more evident at low energies.

**Table 4:** Mean azimuthal angle $\langle \phi \rangle$ of leading, subleading, third jets, and forward dijets at different energies for each generator.

$\sqrt{s}$ (TeV)	Jet rank	PYTHIA8	HERWIG	SHERPA
13.0	Leading	3.135 ± 0.0004551	3.143 ± 0.0004204	3.137 ± 0.001611
13.0	Sublead.	3.146 ± 0.0004564	3.141 ± 0.0004182	3.14 ± 0.001618
13.0	Third	3.136 ± 0.0004562	3.134 ± 0.0004195	3.15 ± 0.00162
13.0	Dijet	3.112 ± 0.001106	3.14 ± 0.0009417	3.172 ± 0.004023
13.6	Leading	3.14 ± 0.000142	3.141 ± 0.0001403	3.142 ± 0.000414
13.6	Sublead.	3.142 ± 0.0001421	3.141 ± 0.0001404	3.137 ± 0.0004136
13.6	Third	3.14 ± 0.000142	3.143 ± 0.0001404	3.142 ± 0.0004135
13.6	Dijet	3.144 ± 0.003415	3.141 ± 0.0003132	3.125 ± 0.001017
14.0	Leading	3.146 ± 0.0004446	3.147 ± 0.0004091	3.131 ± 0.001266
14.0	Sublead.	3.138 ± 0.0004447	3.144 ± 0.0004099	3.138 ± 0.001267
14.0	Third	3.147 ± 0.0004444	3.143 ± 0.0004093	3.146 ± 0.001274
14.0	Dijet	3.130 ± 0.001064	3.148 ± 0.0009112	3.136 ± 0.003156
20.0	Leading	3.142 ± 0.0001268	3.142 ± 0.0001228	3.147± 0.0004594
20.0	Sublead.	3.142 ± 0.0001269	3.142 ± 0.0001229	3.142 ± 0.0004593
20.0	Third	3.141 ± 0.0001268	3.142 ± 0.0001228	3.141 ± 0.0004591
20.0	Dijet	3.146 ± 0.0002865	3.142 ± 0.0002638	3.136 ± 0.0001067
27.0	Leading	3.143 ± 0.0001169	3.141 ± 0.0001095	3.145 ± 0.0003267
27.0	Sublead.	3.14 ± 0.0001168	3.142 ± 0.0001096	3.142 ± 0.0003257
27.0	Third	3.141 ± 0.0001169	3.143 ± 0.0001095	3.139 ± 0.0003256
27.0	Dijet	3.146 ± 0.0002551	3.143 ± 0.0002309	3.146 ± 0.0007318

Uncertainties represent statistical errors.

**Fig. 12:**
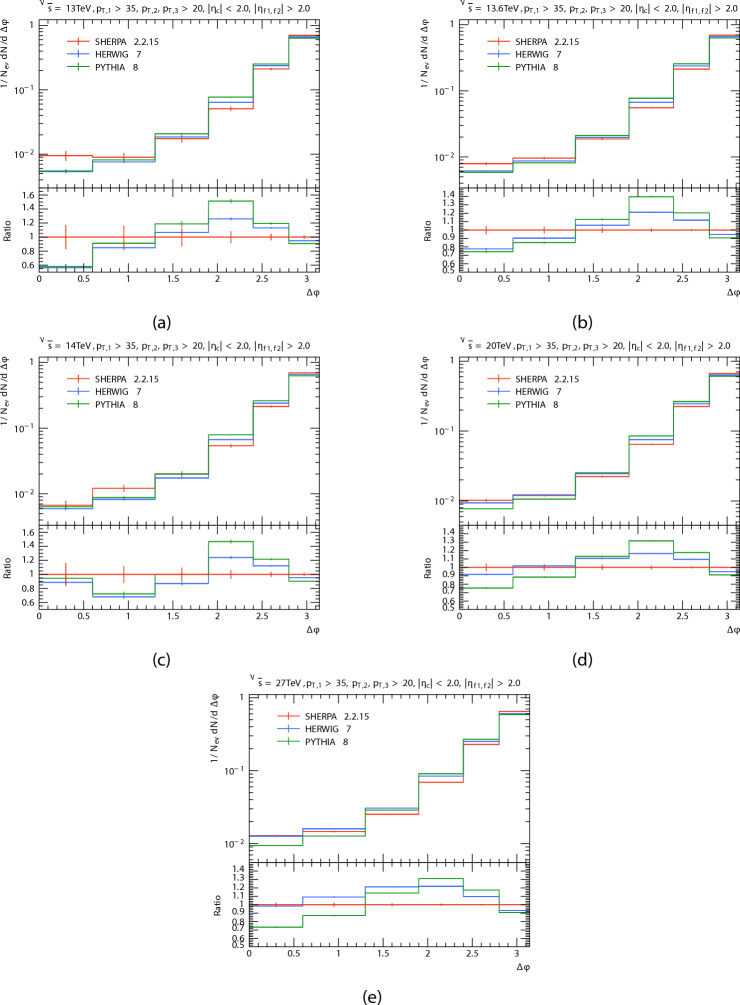
$\Delta \phi$ distributions at 13, 13.6, 14, 20, and 27 TeV, compared PYTHIA, HERWIG with SHERPA predictions.

We compare the predictions of PYTHIA ,HERWIG, and SHERPA at difference energies 13, 13.6, 14, 20 and 27 TeV. The azimuthal angle difference, $\Delta \phi _{dijet}$, between the leading dijet system and the third jet is displayed in Fig. 12. Figure 12a–e aillustrates the predictions at 13, 13.6, 14, 20 and 27 TeV respectively. And the Table 5 shows mean $\Delta \phi$ $\langle \Delta \phi \rangle$ at different energies for each generator. The azimuthal angle difference ($\Delta \phi _{dijet}$) between the third jet and the leading dijet system peaks peaked at $\Delta \phi _{dijet}=\pi$, because of jets produced from hard scattering proceses tend to be emitted in opposite directions due to momentum conservation principles. However, variations across generators at intermediate ($\Delta \phi _{dijet}$) values reflect the different ways they interact with extra radiation and hadronization effects.

It can be observed that as $\Delta \phi _{dijet}$ increases, so does the predicted number of events. It seems to both generators (PYTHIA8 and HERWIG) predict a lower than the SHERPA predicted at low $\Delta \phi$ But when it increases $\Delta \phi$, (PYTHIA8 and HERWIG) predictions are higher than SHERPA prediction. But when the energy increases, the predictions of the HERWIG Approaching that of the SHERPA at low $\Delta \phi.$

**Table 5:** Mean $\Delta \phi$ $\langle \Delta \phi \rangle$ at different energies for each generator.

$\sqrt{s}$ (TeV)	PYTHIA8	HERWIG	SHERPA
13.0	2.755 ± 0.0008961	2.775 ± 0.0007333	2.783 ± 0.003322
13.6	2.751 ± 0.0002797	2.766 ± 0.00023537	2.78 ± 0.000411
14.0	2.747 ± 0.0008825	2.771 ± 0.0007212	2.777 ± 0.002603
20.0	2.726 ± 0.0002565	2.734 ± 0.0002428	2.752 ± 0.0009702
27.0	2.706 ± 0.000244	2.7 ± 0.000236+	2.73 ± 0.0007205

Uncertainties represent statistical errors.

**Fig. 13:**
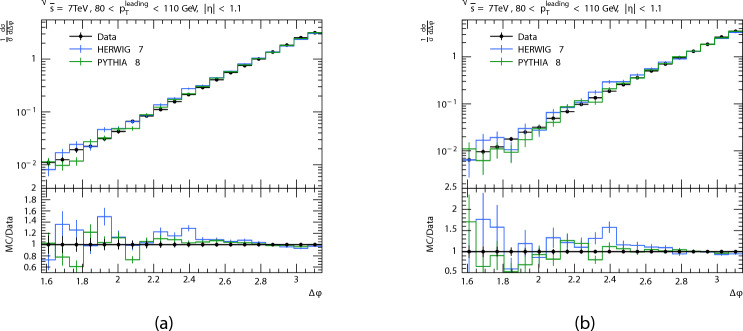
$\Delta \phi$ distributions at 7 TeV PYTHIA8 and HERWIG simulations is compared with the results from CMS^28^.

We also compare cross section of the PYTHIA8 and HERWIG predictions with with the real data of CMS^28^ as a function of $\Delta \phi$ at energy 7 TeV with The azimuthal angle difference, $\Delta \phi$, between the leading jet and the subleading jet is displayed in Fig. 13. It can be observed that as $\Delta \phi$ increases, so does the cross section. The azimuthal angle ($\Delta$) also peaks at $\Delta \phi _{dijet}=\pi$, because of jets produced from hard scattering processes tend to be emitted in opposite directions due to momentum conservation principles. We notice that in the first region$80<p_{T}^\textrm{leading}<110\,\hbox {GeV}$, tPYTHIA shows agreement with the data and HERWIG’s prediction is less than the data, then they decrease from the data for PYTHIA8, and for HERWIG it starts to increase over data. And there is a fairly good agreement in the high values of $\Delta \phi$ for both generators. As for the second region $110<p_{T}^\textrm{leading}<140\,\hbox {GeV}$, when the $p_T$ values increase, the difference between the data and the predictions increases at the beginning, and this difference is higher for HERWIG, and there is an agreement in the high values of $\Delta \phi.$

It is observed that different generators disagree at some data points and that some histograms have too much data. This be because various theoretical models are used to each event generator. The PYTHIA model utilizes the Lund string model for hadronization, resulting in a more extensive jet structure during multi-jet events, and employs a transverse-momentum-ordered parton shower model that effectively simulates parton radiation and fragmentation, particularly managing soft emissions and underlying event dynamics. In contrast, HERWIG uses an angular-ordered parton shower algorithm that produces narrower jets and employs a cluster hadronization model, enhancing its accuracy for next-to-leading order (NLO) processes and making it suitable for precision measurements in collider experiments. SHERPA distinguishes itself by automating the merging of leading-order matrix elements with parton showers, effectively simulating high jet multiplicities and complex event topologies while also using a cluster hadronization model.

## Conclusions

In the present work, jet generation predictions for proton-proton collisions at center-of-mass energies of 13, 13.6, 14, 20 and 27 TeV were compared between the PYTHIA8, HERWIG, and SHERPA Monte Carlo event generators. The event with 3 jets We concentrated on events including three jets or more, with two of the jets located in the forward region and one in the middle of the pseudorapidity region. The distributions of the jet multiplicity, azimuthal angle $(\phi )$, transverse momentum $(p_T)$, and pseudorapidity $(\eta )$ were studied.

This study reveals significant insights into the performance of different event generators. The results indicate that as the center-of-mass energy increases, the jet multiplicity distributions and the number of jets produced also increases. Additionally, phase limitations in space and decreasing probability of finding high momentum partons within protons are the causes of the multiplicities’ rapid decrease with increasing $p_T$. Additionally, the pseudorapidity distributions reveal that central regions exhibit greater jet multiplicity due to more efficient parton interactions. And all generators showing good agreement in central regions but diverging at the extremes. The predictions have a quasi-flat distribution, there are some fluctuations in the predictions that are more noticeable in SHERPA and HERWIG’s distribution throughout all regions. And it is observed that a peak at $\Delta \phi _{dijet}=\pi$ for all generators when the azimuthal angle difference ($\Delta \phi _{dijet}$) due to momentum conservation principles. The comparison between PYTHIA8 and HERWIG predictions and CMS data at 7 TeV reveals important trends. The cross section increases with $\Delta \phi$ and peaks near $\pi$, consistent with the expectation from momentum conservation in dijet events. In the lower $p_T$ region, the PYTHIA8 and HERWIG shows deviation with data at small $\Delta \phi$, but shows good agreement at higher values. In the higher $p_T$ region , the deviation between predictions and data becomes more pronounced at small angles, and especially for HERWIG, although convergence is again observed at larger $\Delta \phi$. These observations highlight the sensitivity of azimuthal correlations to QCD modeling. Overall, the different generators disagree at some data points and that some histograms have too much data. This is because various theoretical models are used for each event generator.

These findings highlight the need for more theoretical refinement and experimental validation of event generators. Despite having several important characteristics in common, the generators’ differences in high-multiplicity, high-$p_T$, and forward jet production indicate regions that need more theoretical development and experimental validation. Future research should include comprehensive comparisons with LHC experimental data to fine-tune generating parameters. Furthermore, extending the study to next-to-leading-order (NLO) computations and including higher-order QCD effects may improve the precision of predictions. Understanding these discrepancies is critical for developing Monte Carlo simulations, which are used in high-energy physics analysis and novel physics searches at the LHC and future colliders.

## Data Availability

The codes used during the current study available at the following link: https://github.com/maboelyouser/ Trijet
